# Supplementary material for: Design and Synthesis of Immunoadjuvant QS-21 Analogs and Their Biological Evaluation
Source: Biomedicines. 2024 Feb 19;12(2):469. doi: 10.3390/biomedicines12020469 (PMC10887094; doi:10.3390/biomedicines12020469)
Supplement: Supplementary file 1 [file biomedicines-12-00469-s001.zip › biomedicines-2737240-supplementary.pdf]

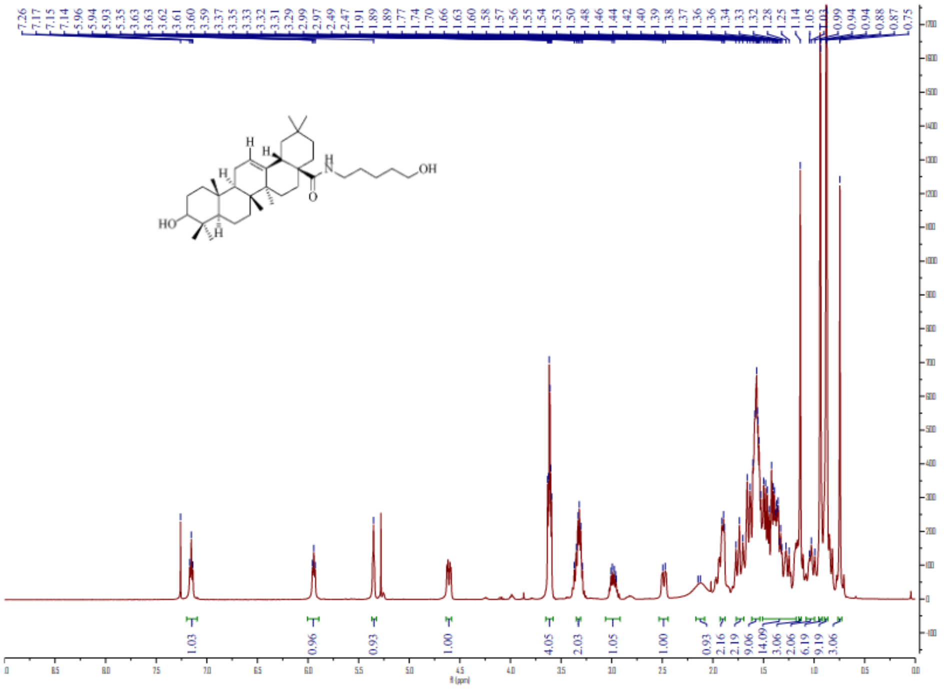

Figure S1. ^{1}H NMR of L1

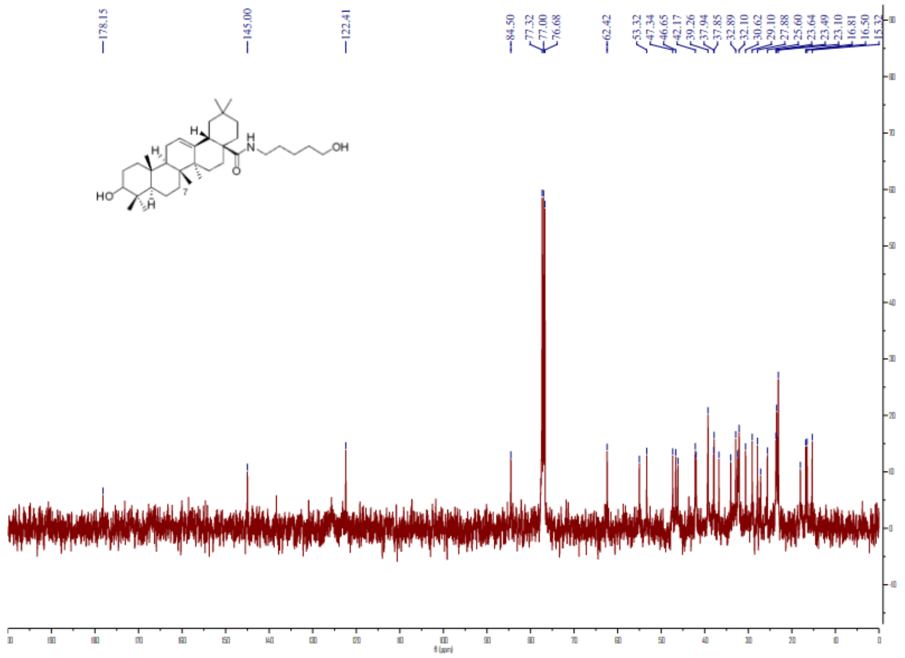

Figure S2. ^{13}C NMR of L1

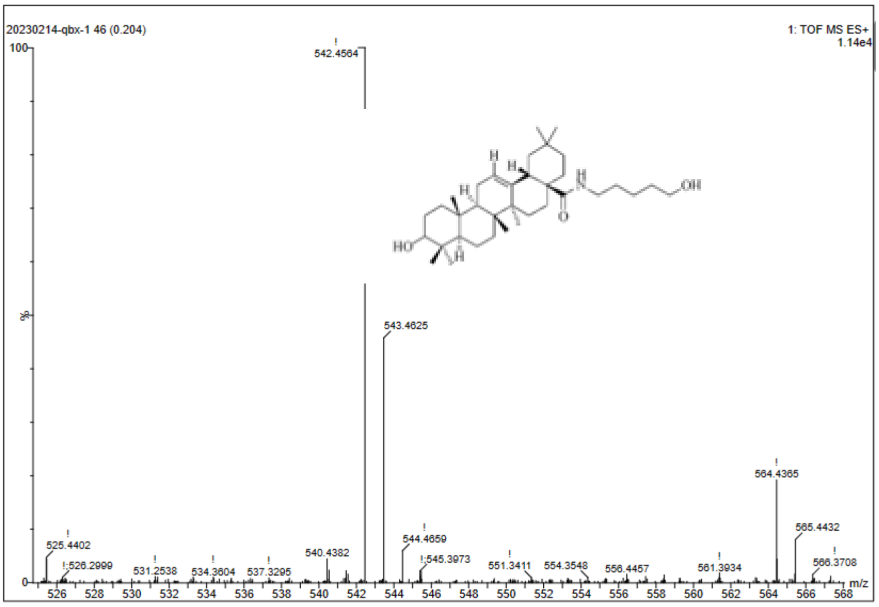

Figure S3. HRMS of L1 (ESI+)

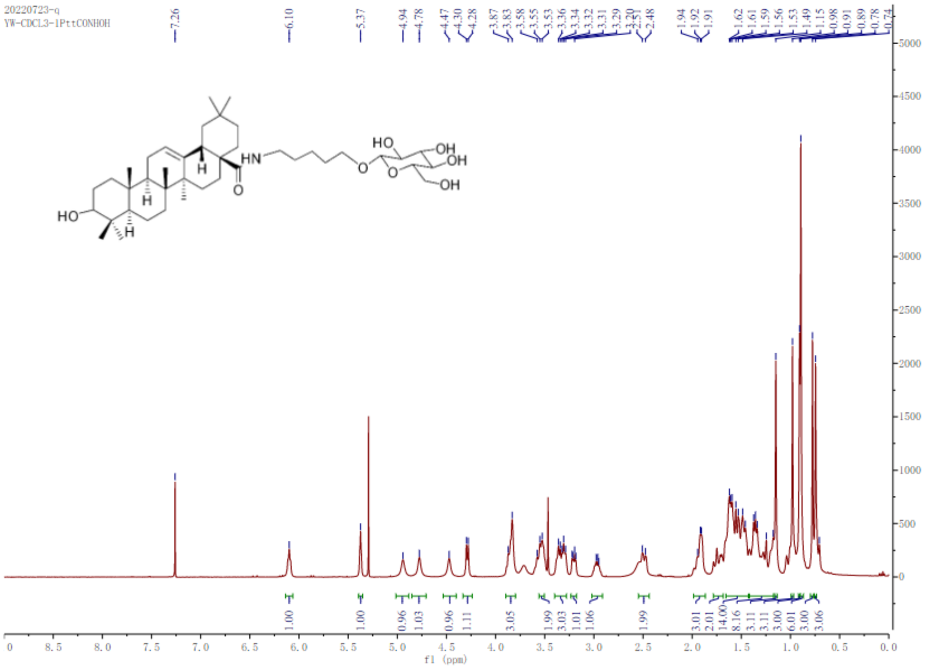

Figure S4. ^{1}H NMR of L2

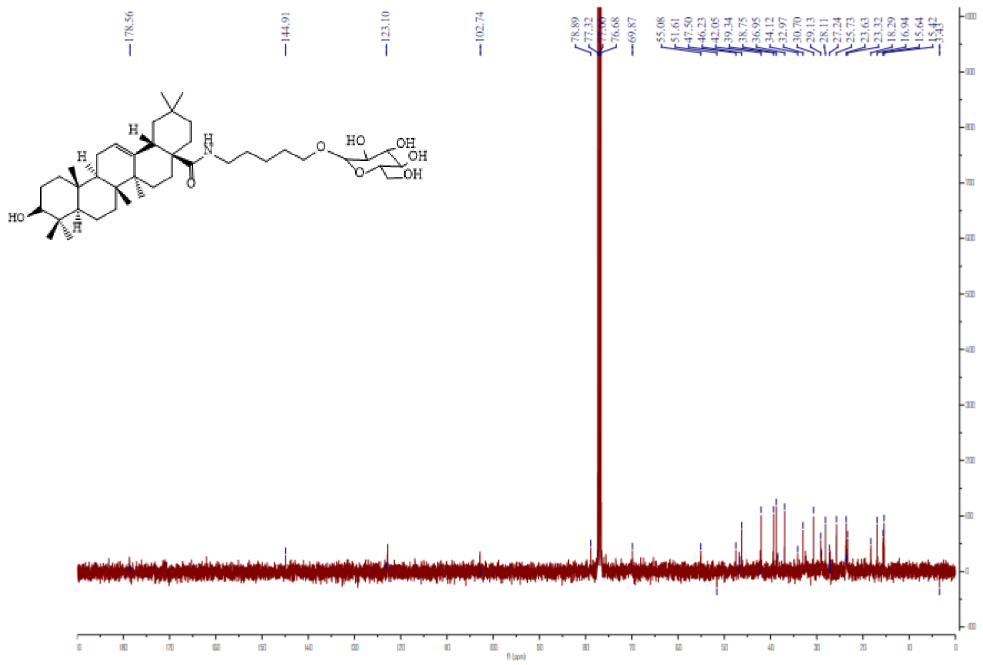

Figure S5. ^{13}C NMR of L2

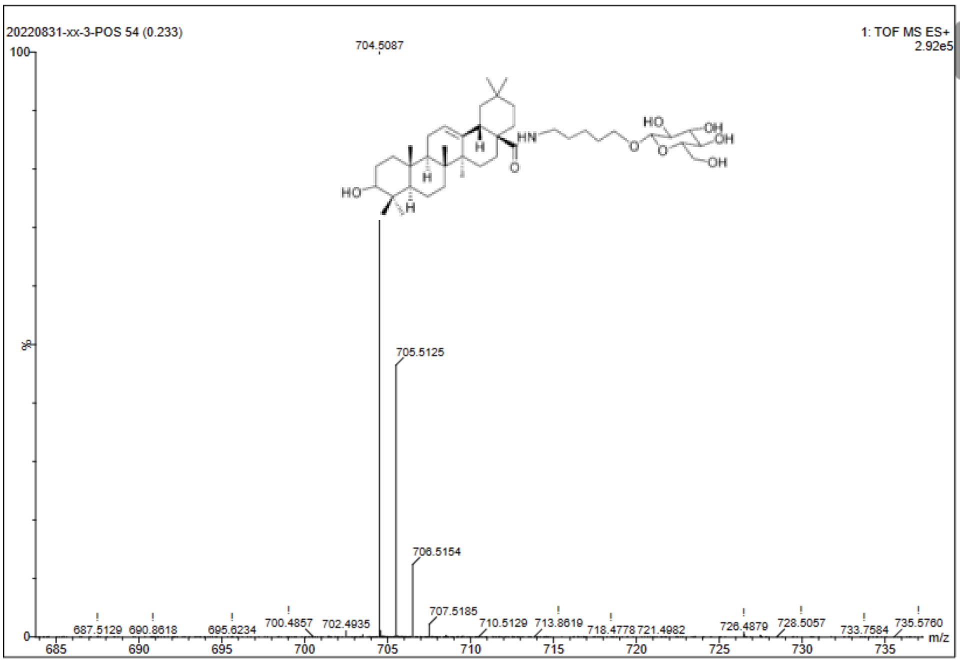

Figure S6. HRMS of L2 (ESI+)

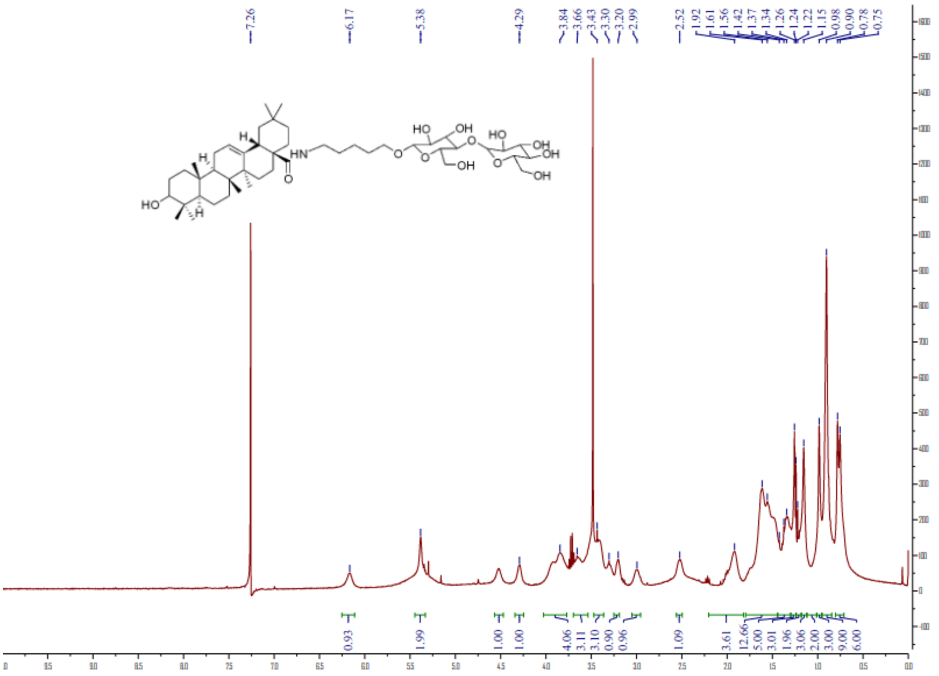

Figure S7. ^{1}H NMR of L3

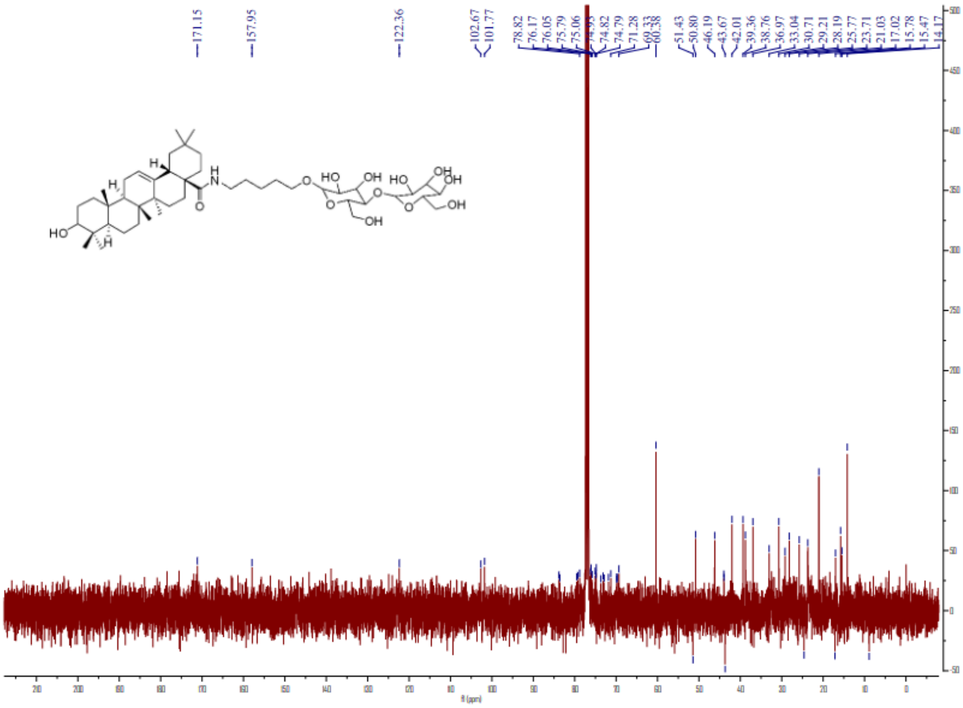

Figure S8. ^{13}C NMR of L3

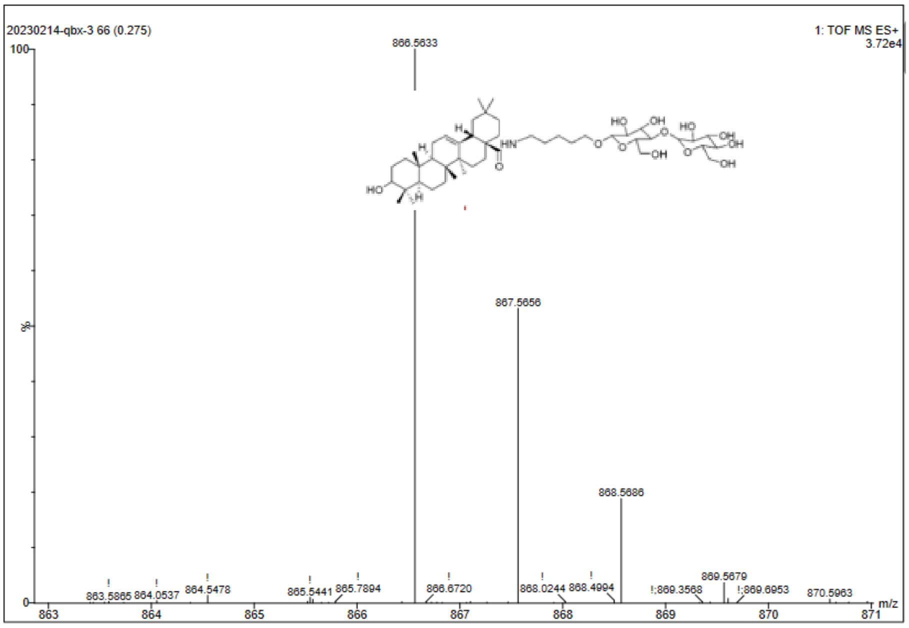

Figure S9. HRMS of L3 (ESI+)

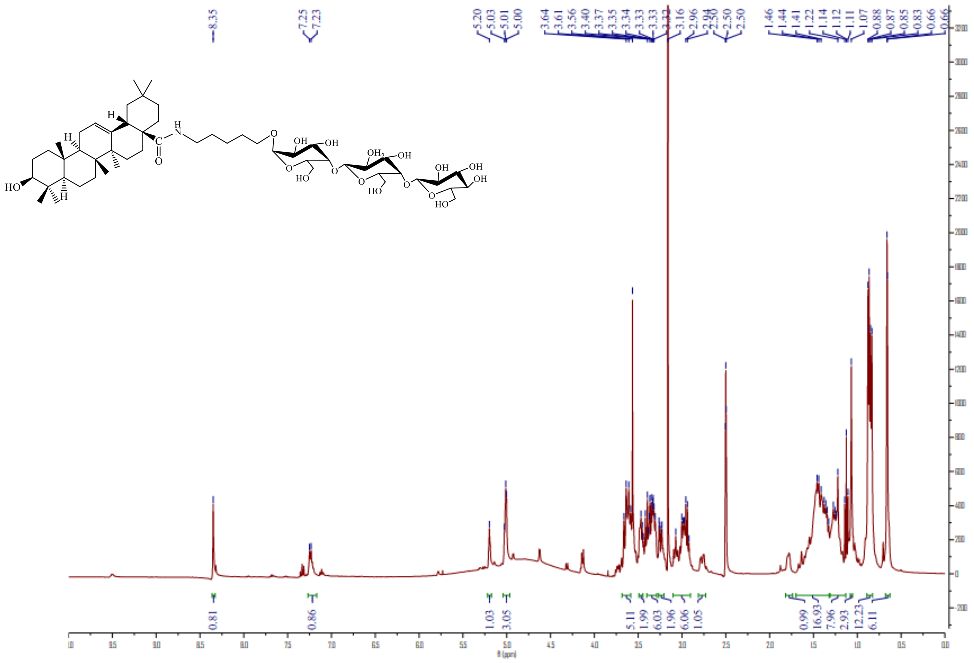

Figure S10. ^{1}H NMR of L4

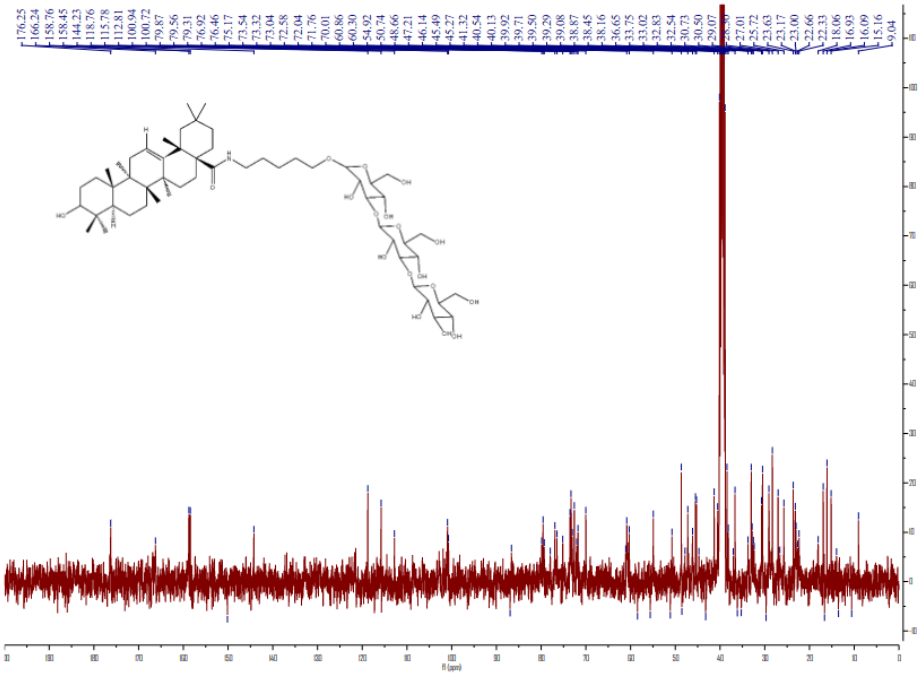

Figure S11. ^{13}C NMR of L4

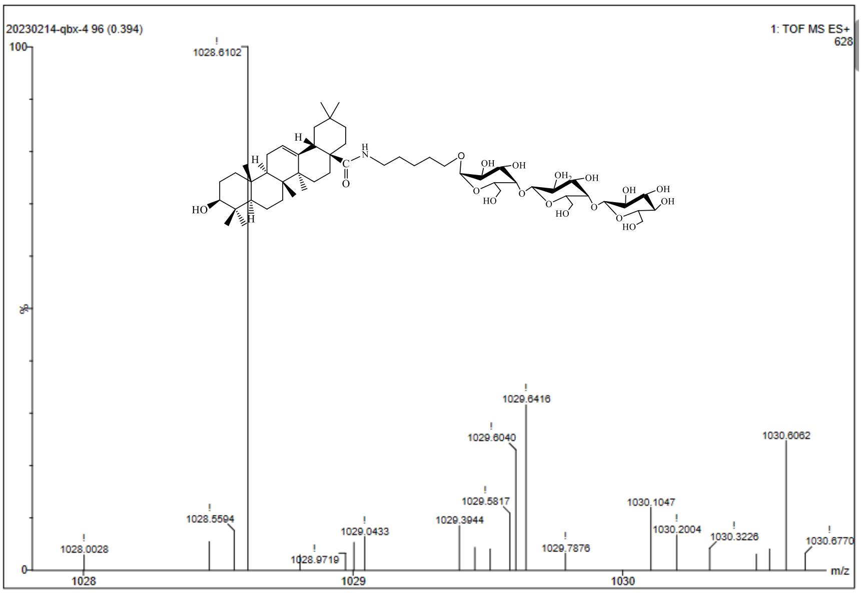

Figure S12. HRMS of L4 (ESI+)

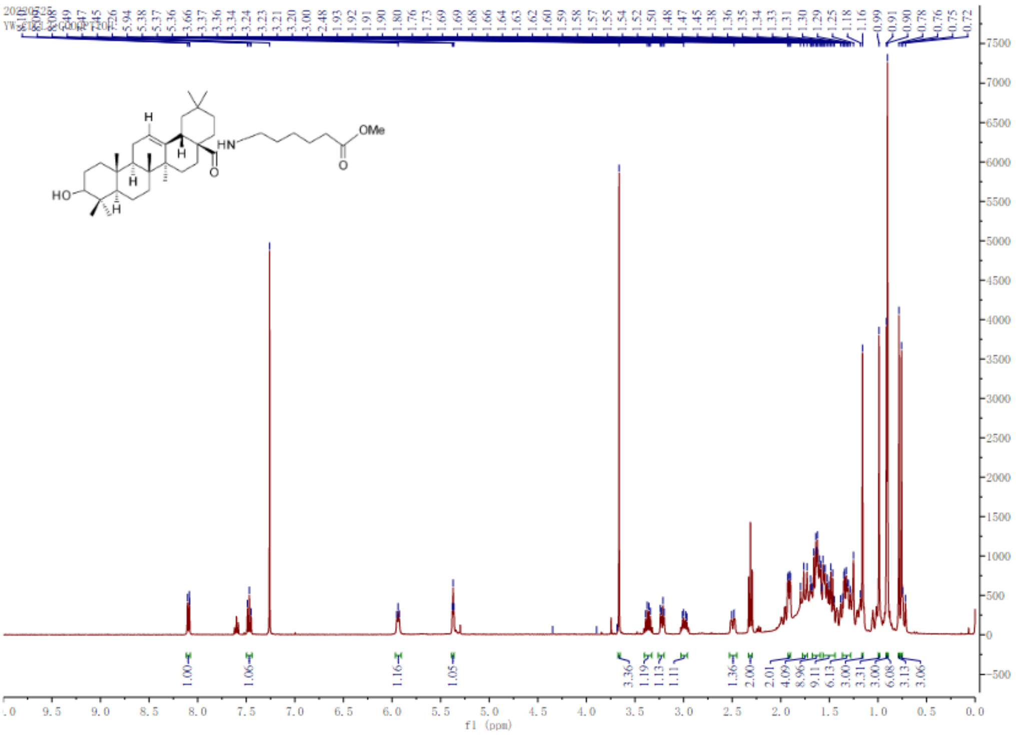

Figure S13. ^{1}H NMR of L5

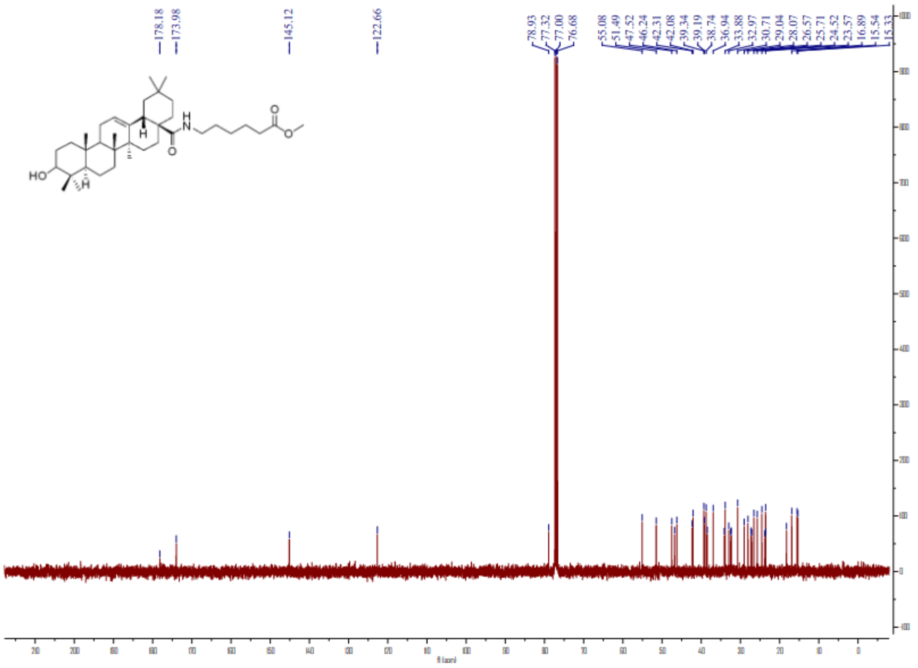

Figure S14. ^{13}C NMR of L5

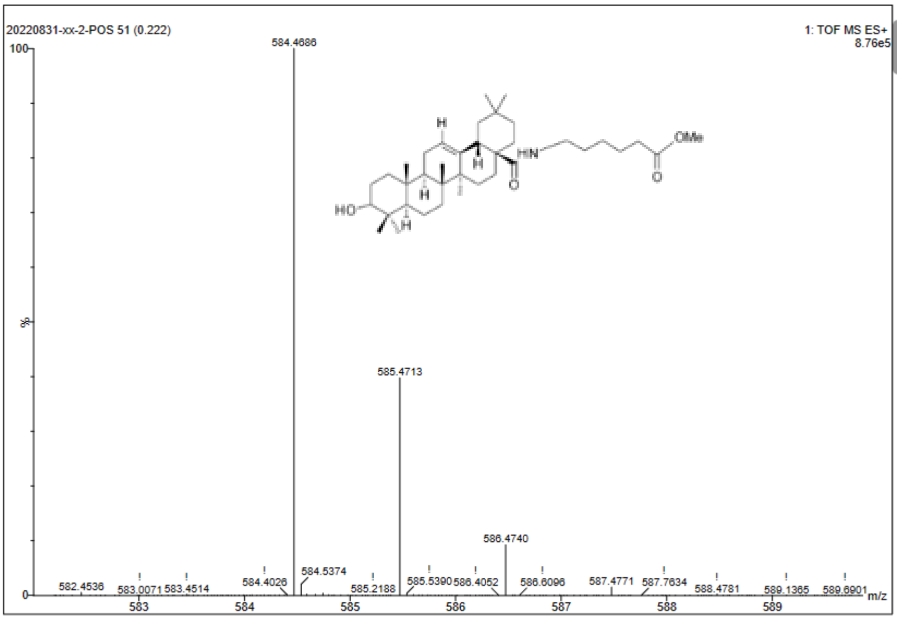

Figure S15. HRMS of L5 (ESI+)

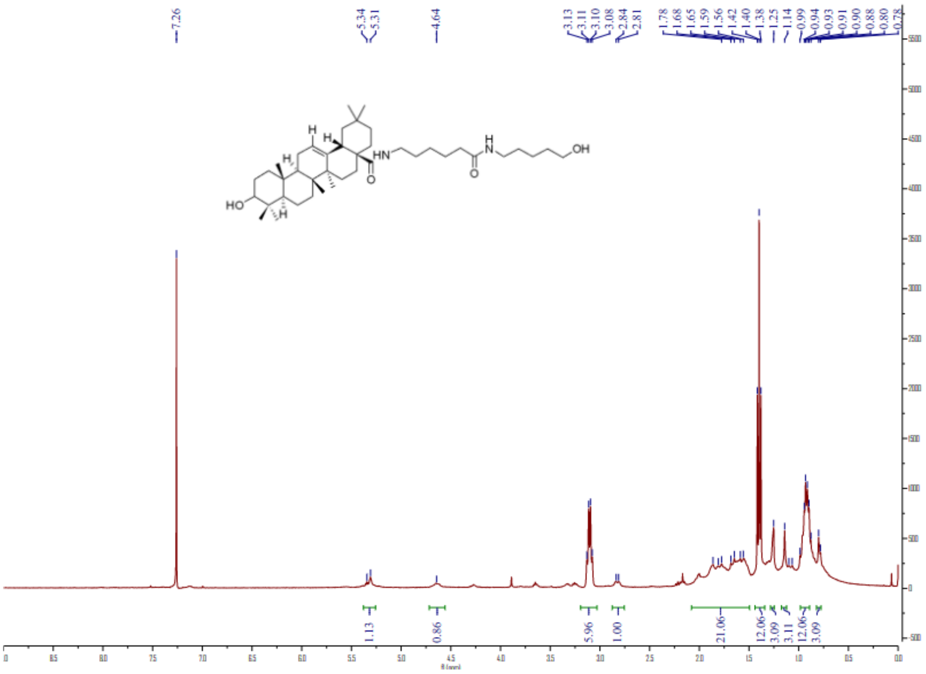

Figure S16. ^{1}H NMR of L6

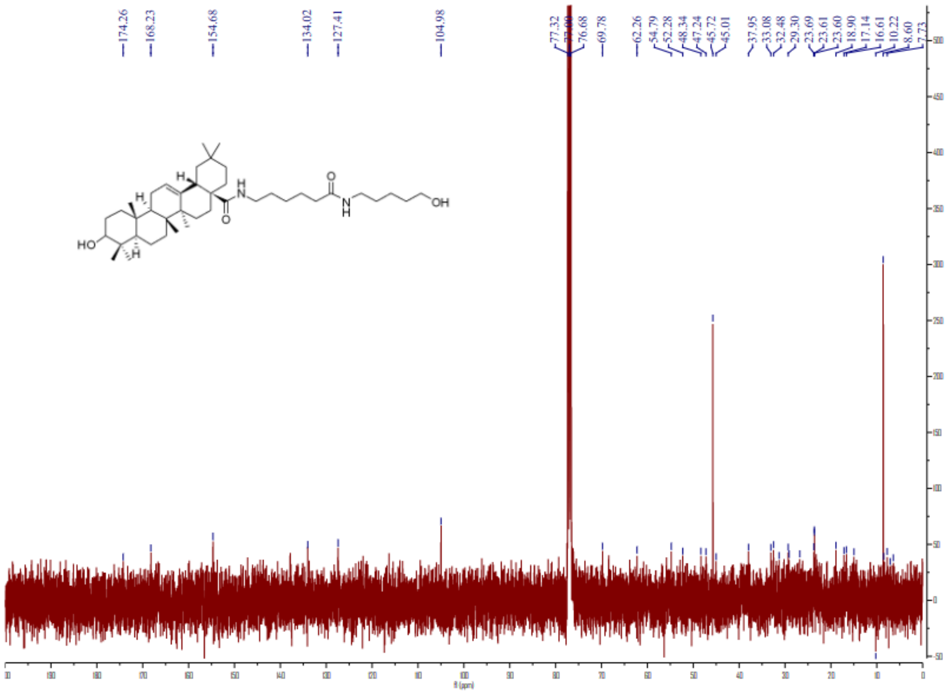

Figure S17. ^{13}C NMR of L6

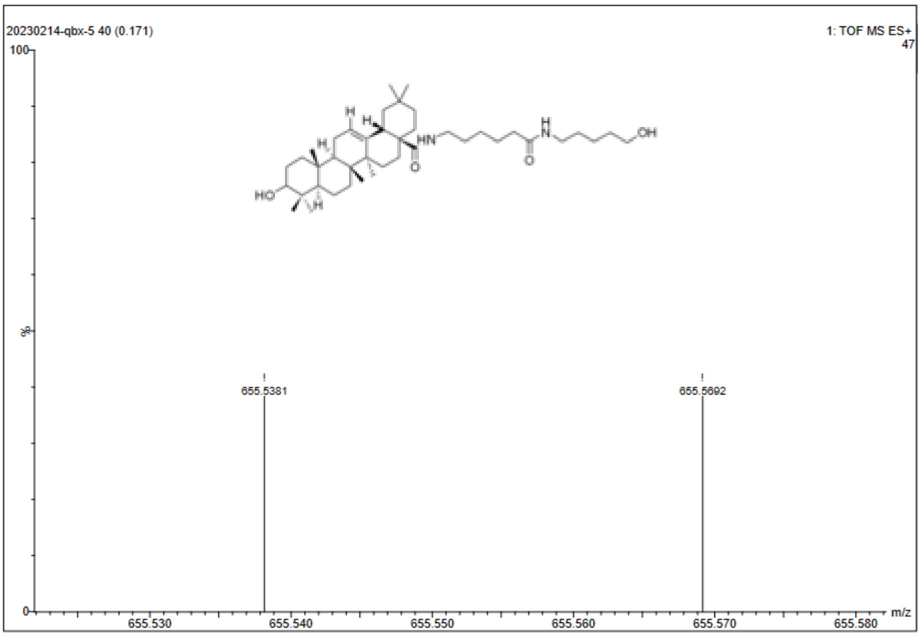

Figure S18. HRMS of L6 (ESI+)

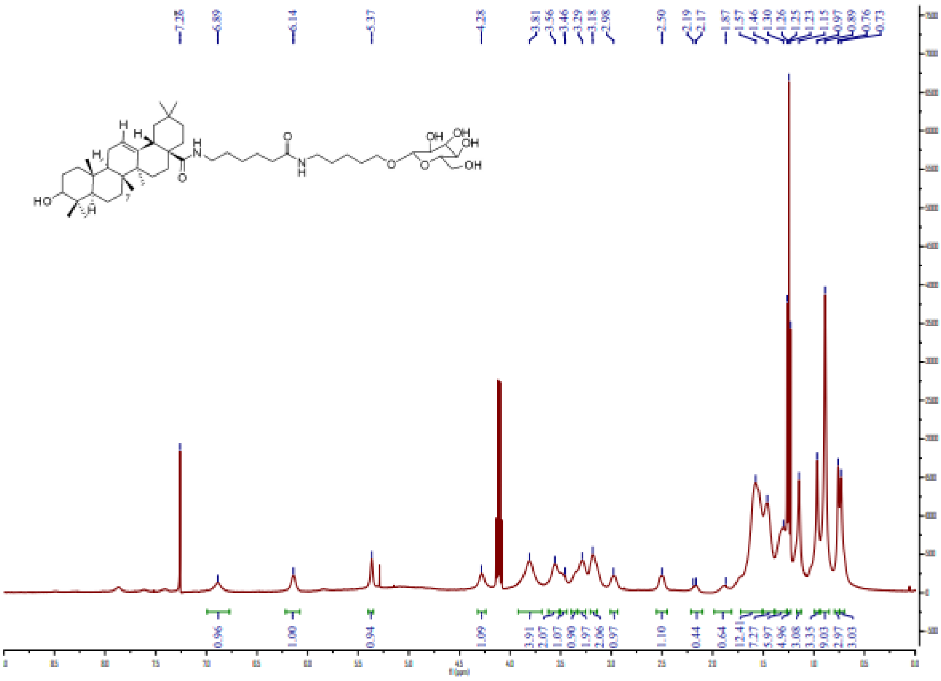

Figure S19. ^{1}H NMR of L7

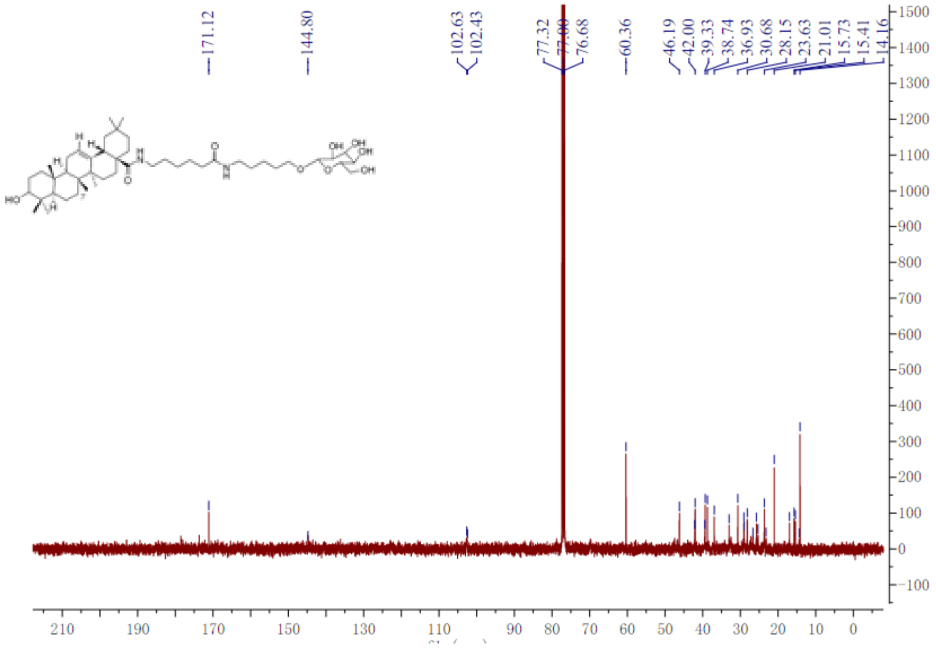

Figure S20. ^{13}C NMR of L7

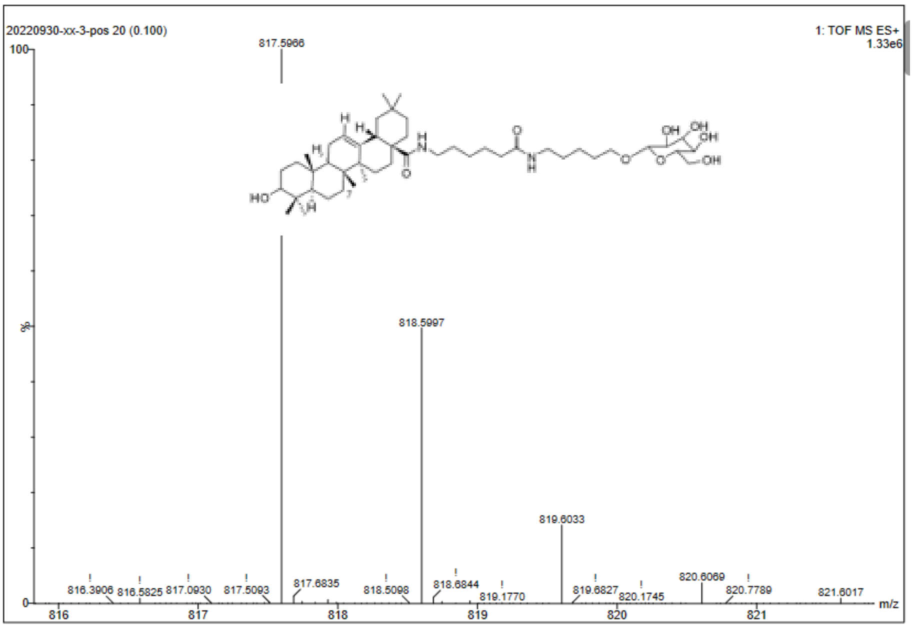

Figure S21. HRMS of L7 (ESI+)

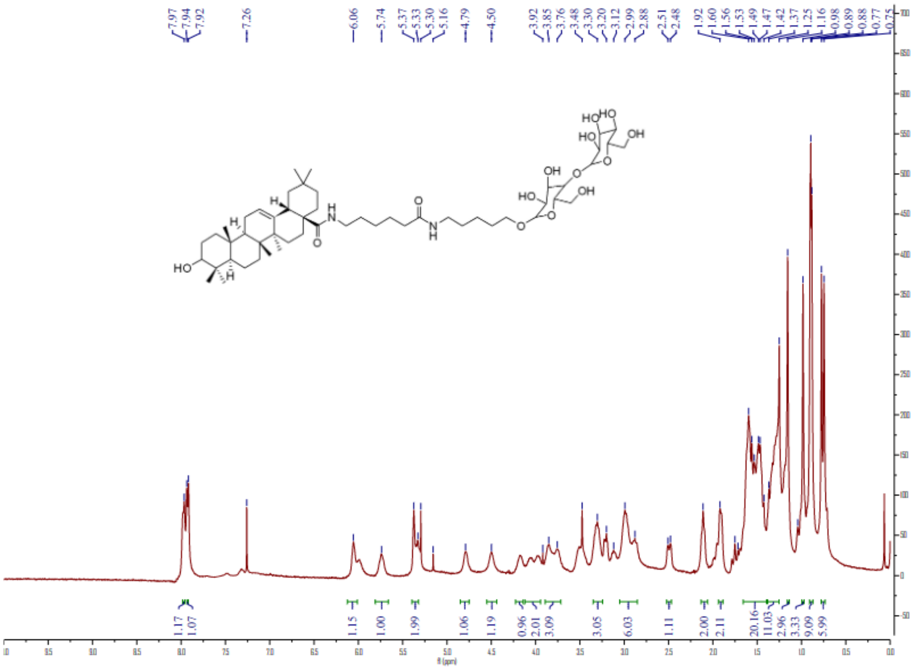

Figure S22. ^{1}H NMR of L8

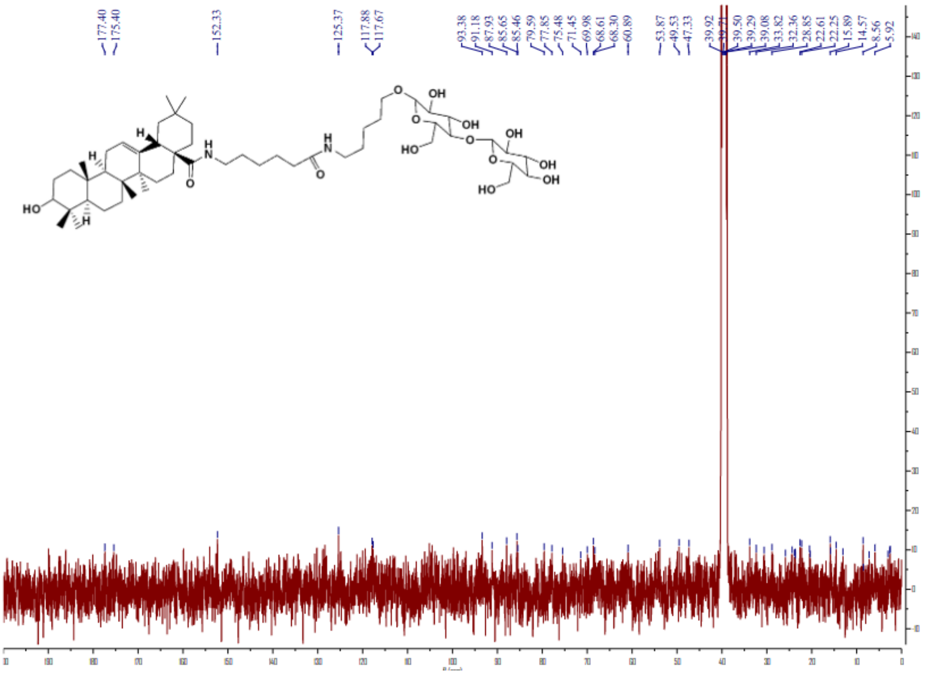

Figure S23. ^{13}C NMR of L8

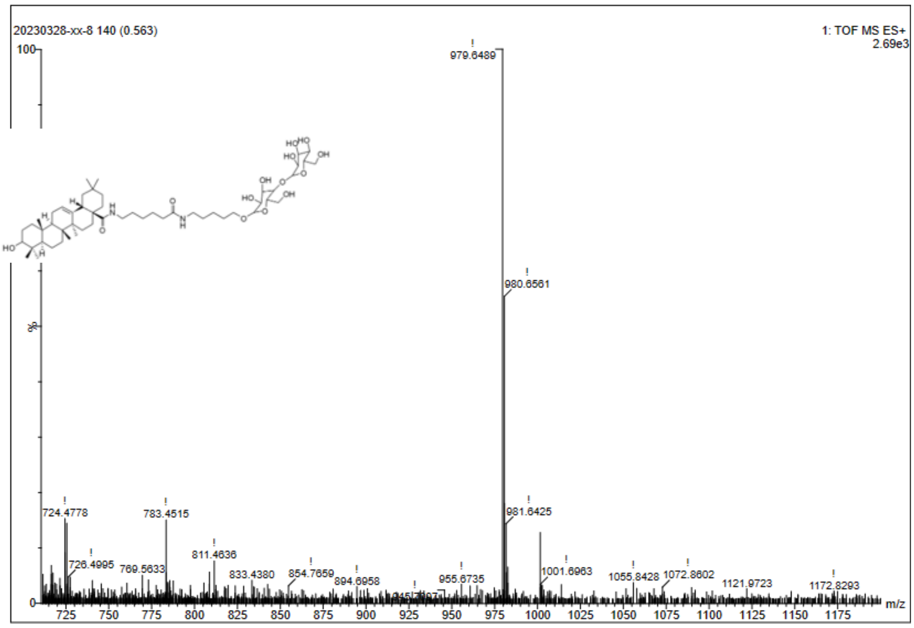

Figure S24. HRMS of L8 (ESI$^+$)

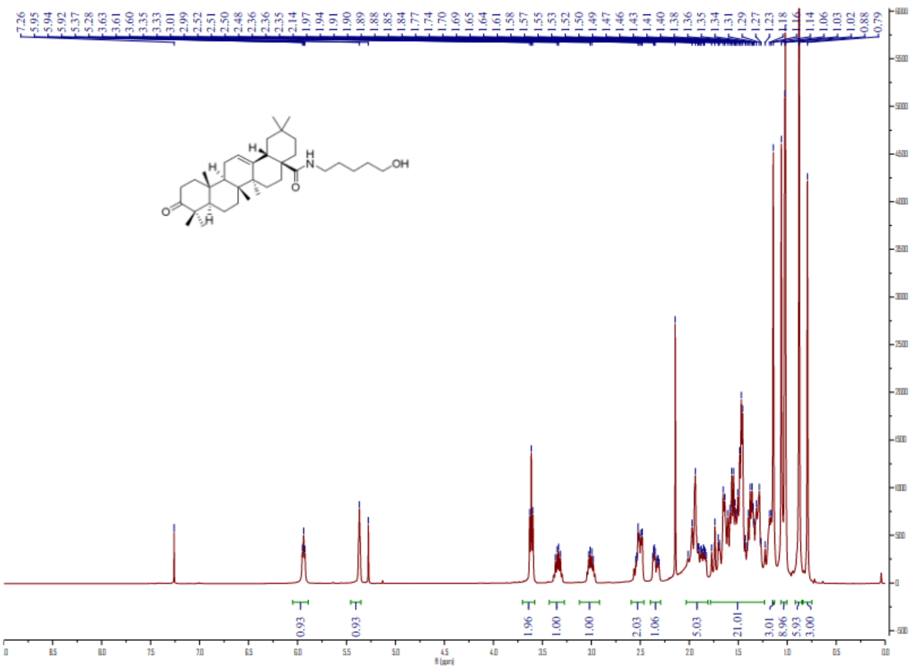

Figure S25. ^{1}H NMR of L9

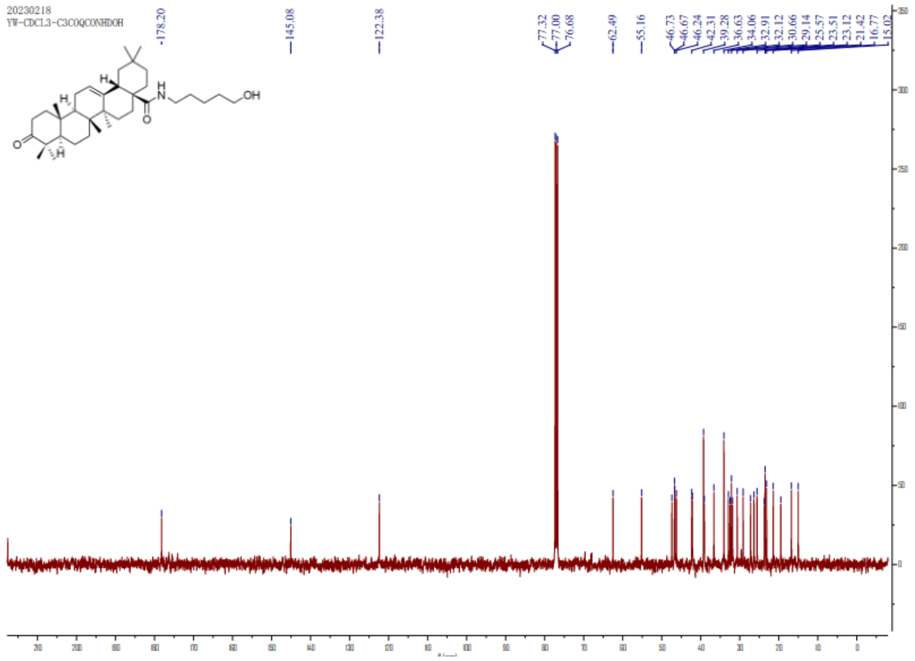

Figure S26. ^{13}C NMR of L9

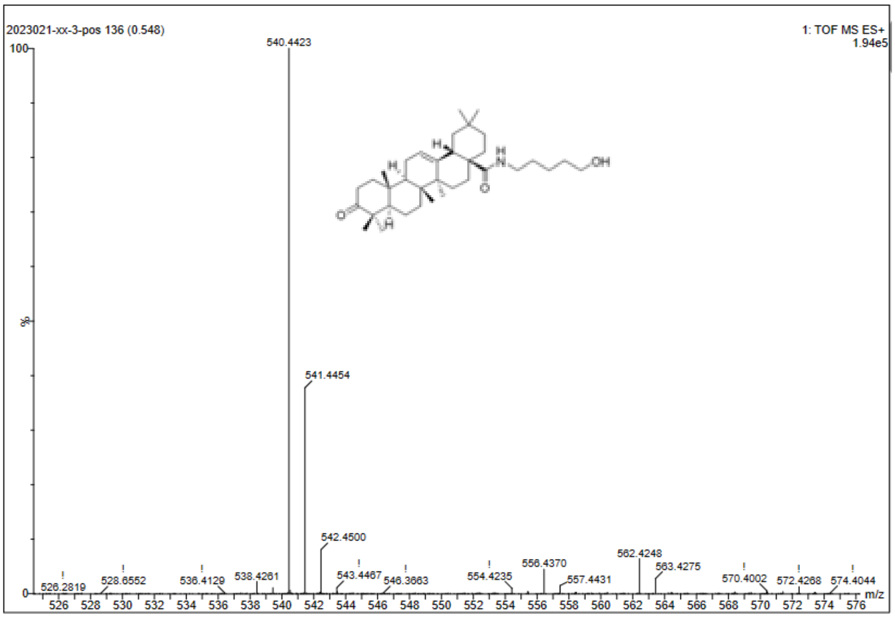

Figure S27. HRMS of L9 (ESI$^+$)

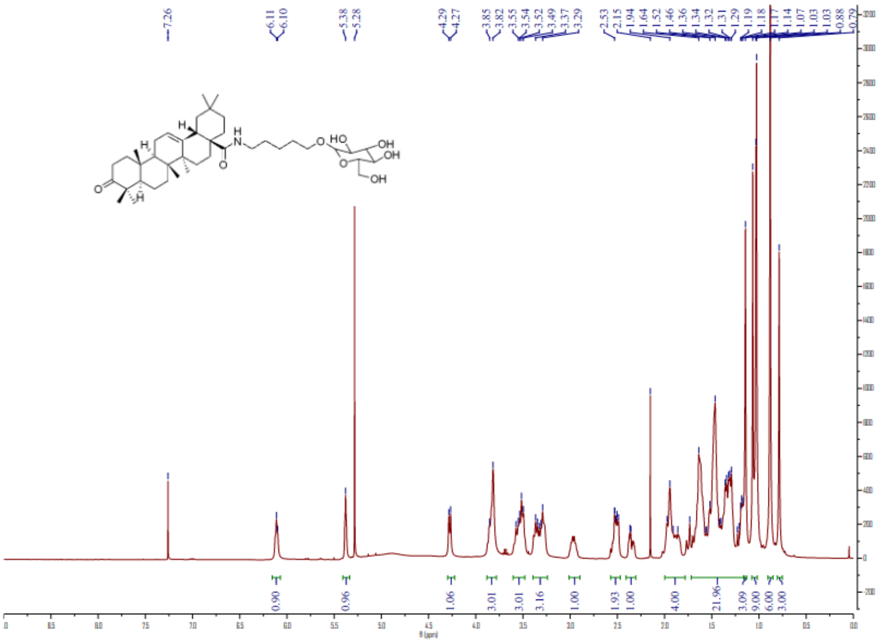

Figure S28. ^{1}H NMR of L10

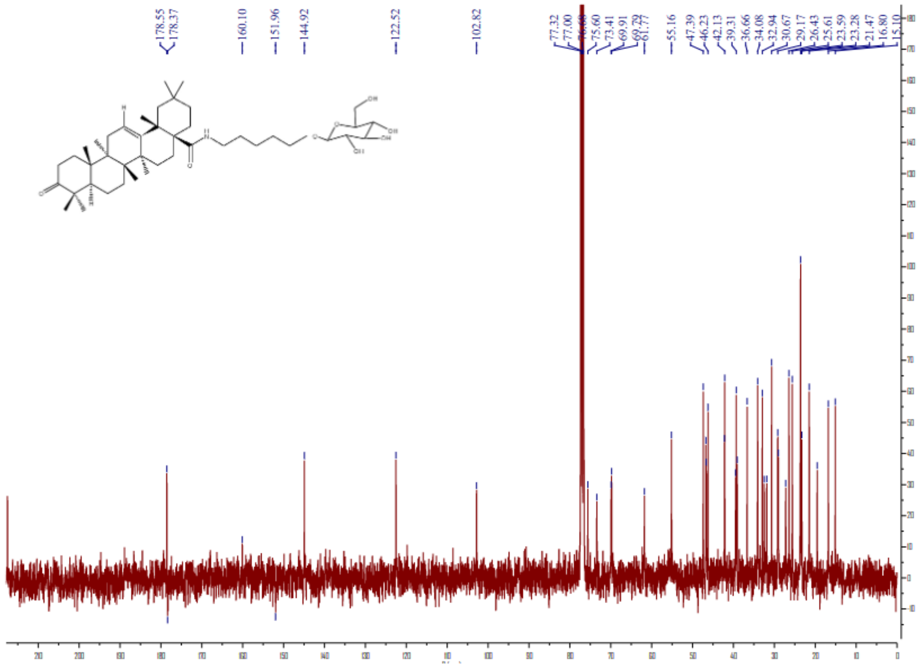

Figure S29. ^{13}C NMR of L10

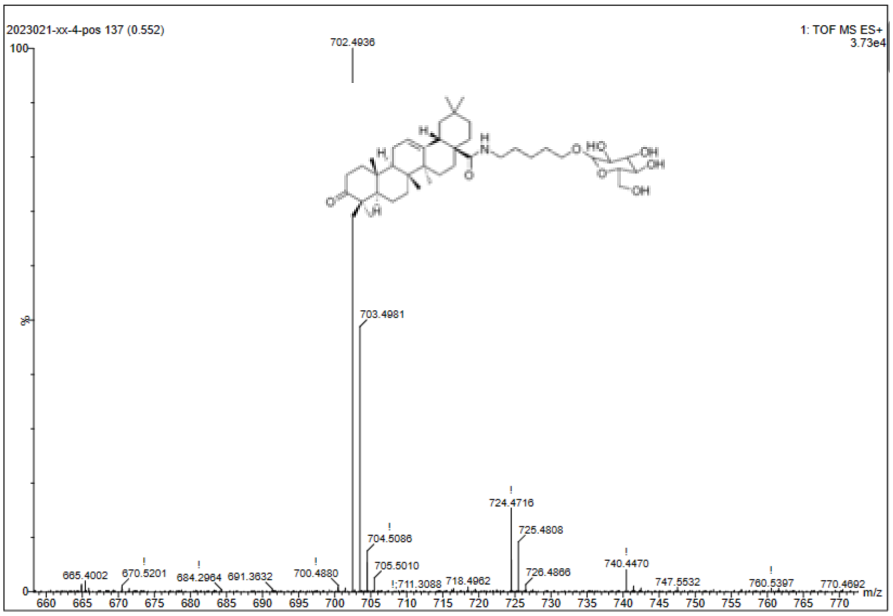

Figure S30. HRMS of L10 (ESI+)

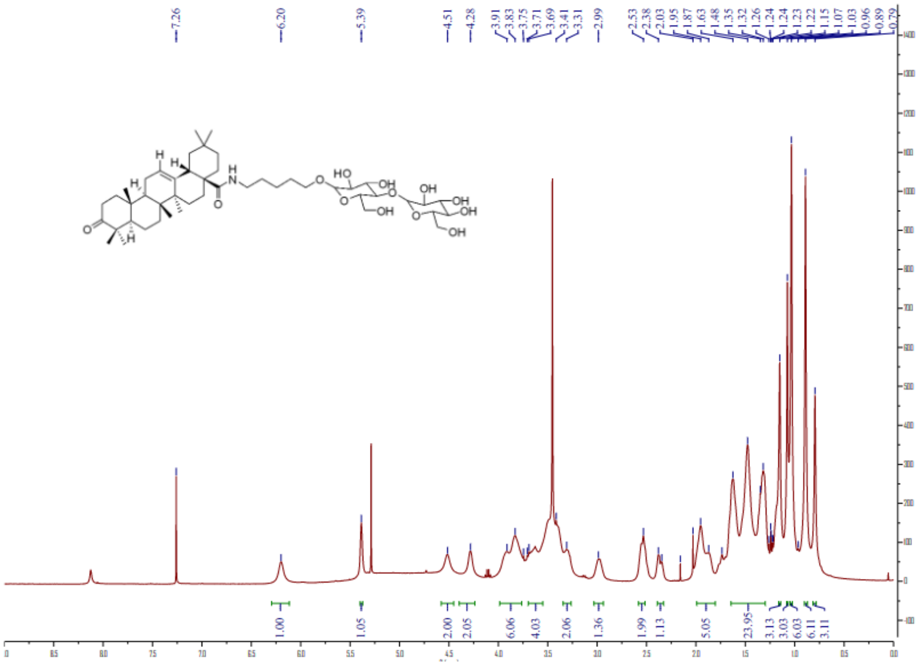

Figure S31. ^{1}H NMR of L11

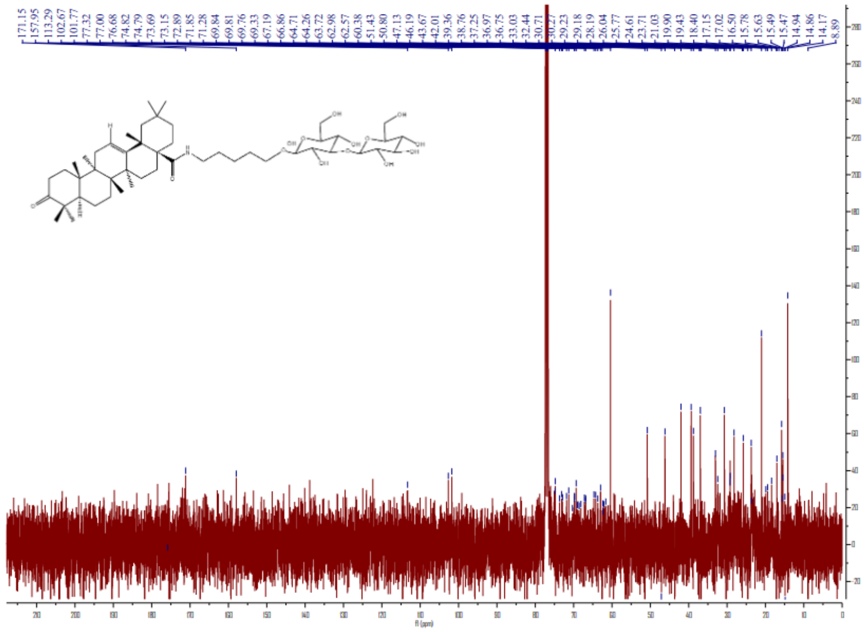

Figure S32. ^{1}H NMR of L11

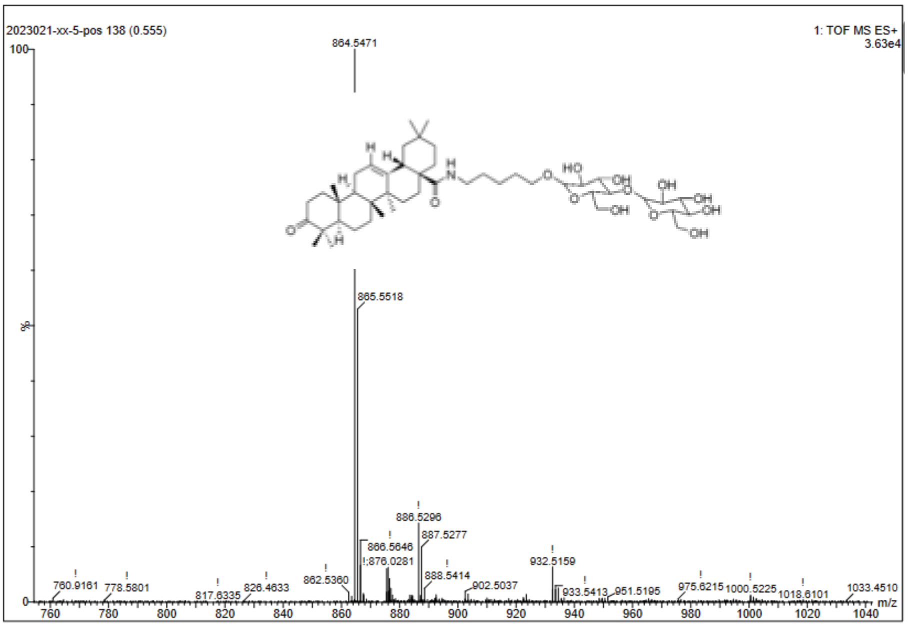

Figure S33. HRMS of L11 (ESI+)

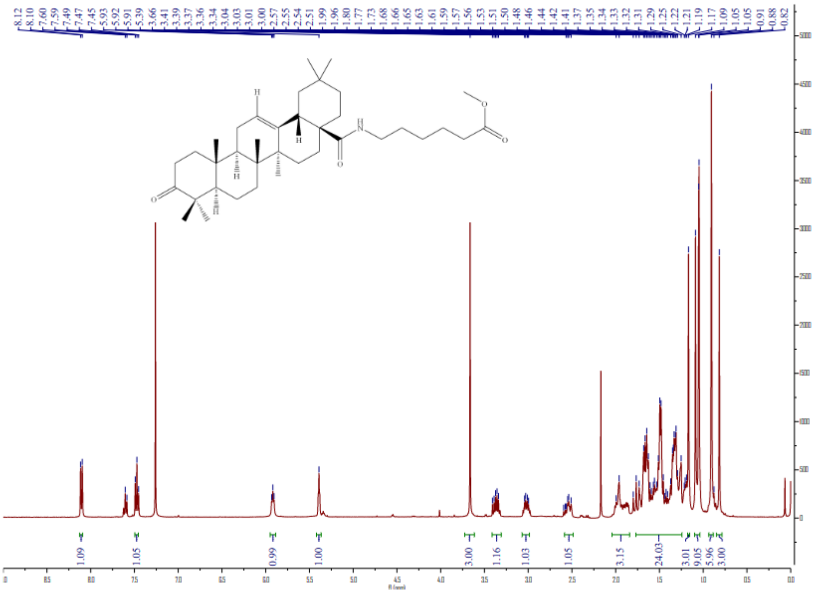

Figure S34. ^{1}H NMR of L12

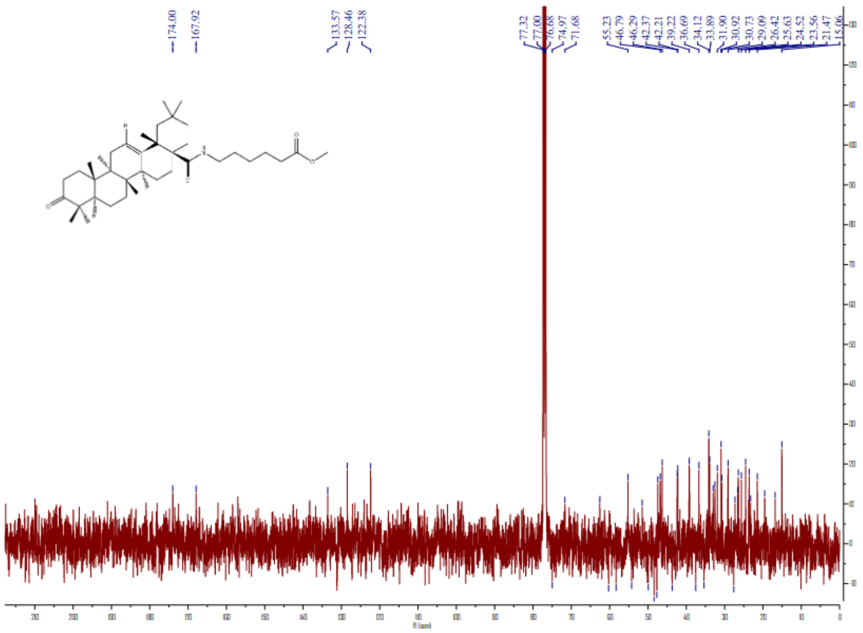

Figure S35. ^{13}C NMR of L12

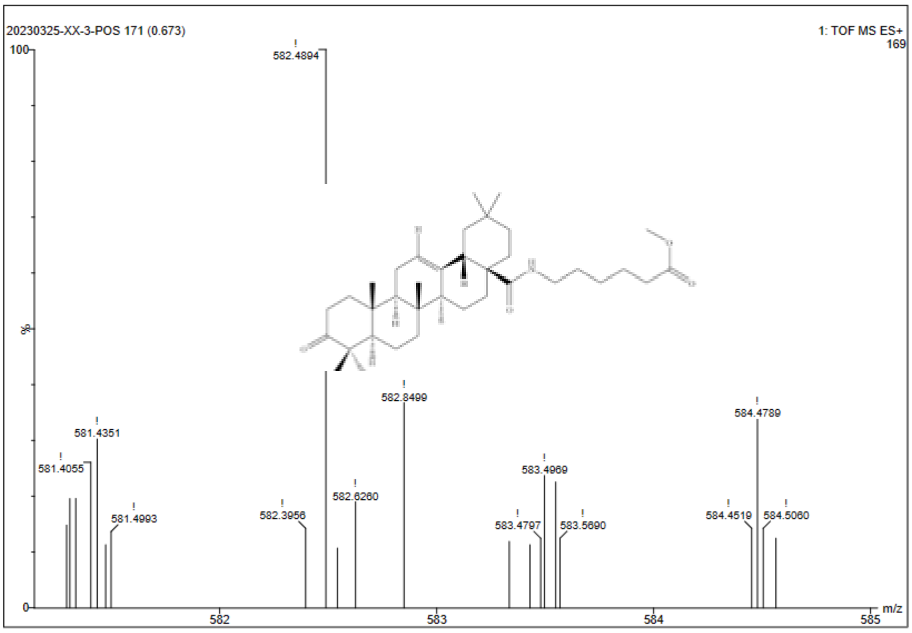

Figure S36. HRMS of L12 (ESI+)

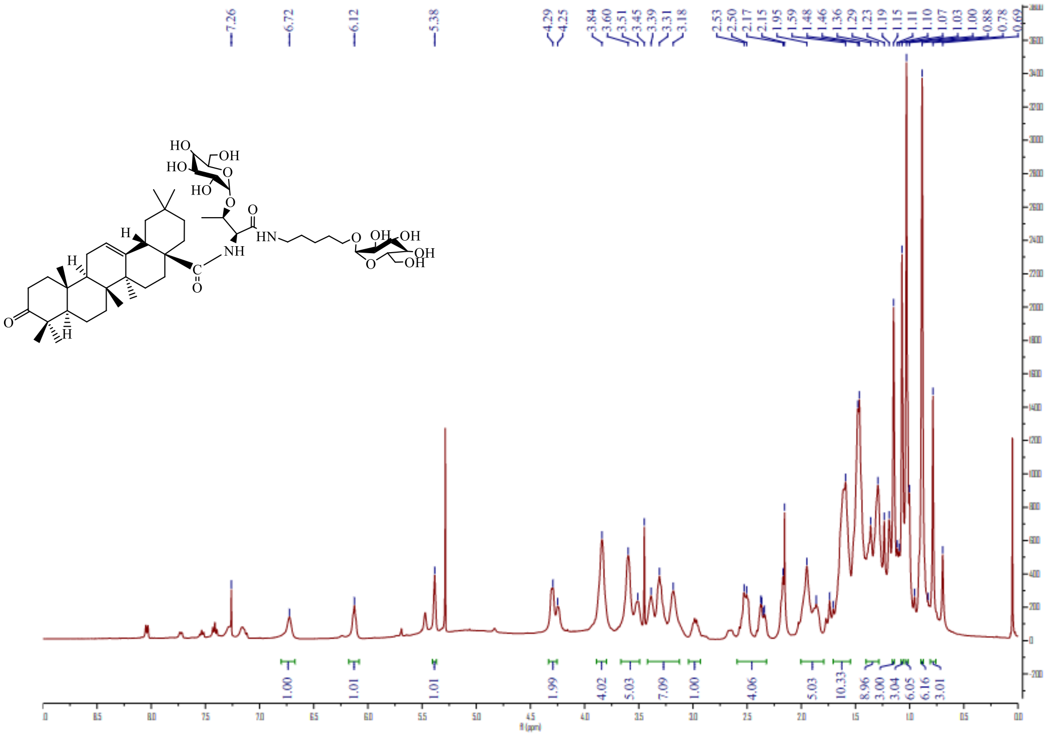

Figure S37. ^{1}H NMR of L13

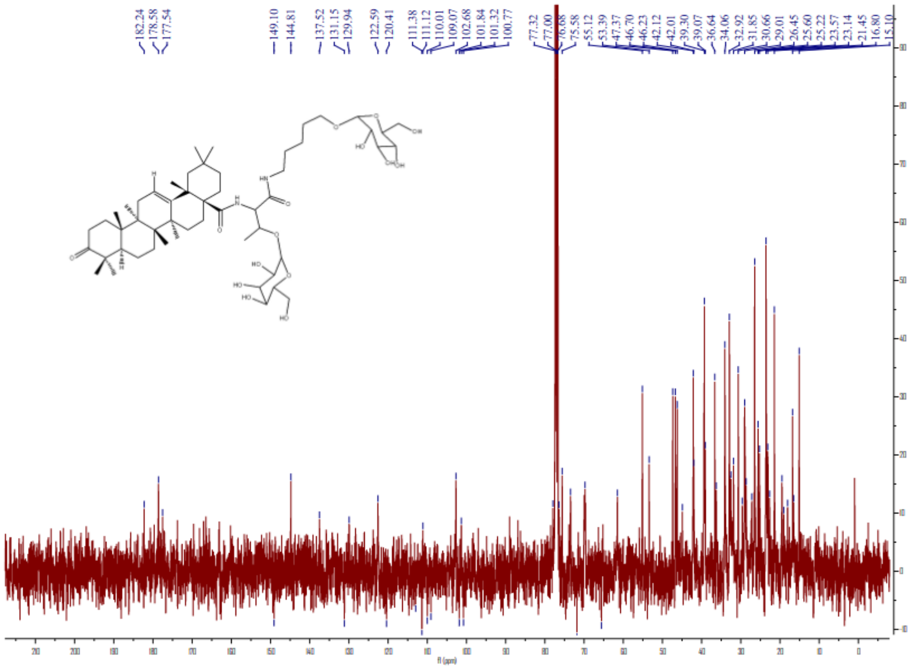

Figure S38. ^{13}C NMR of L13

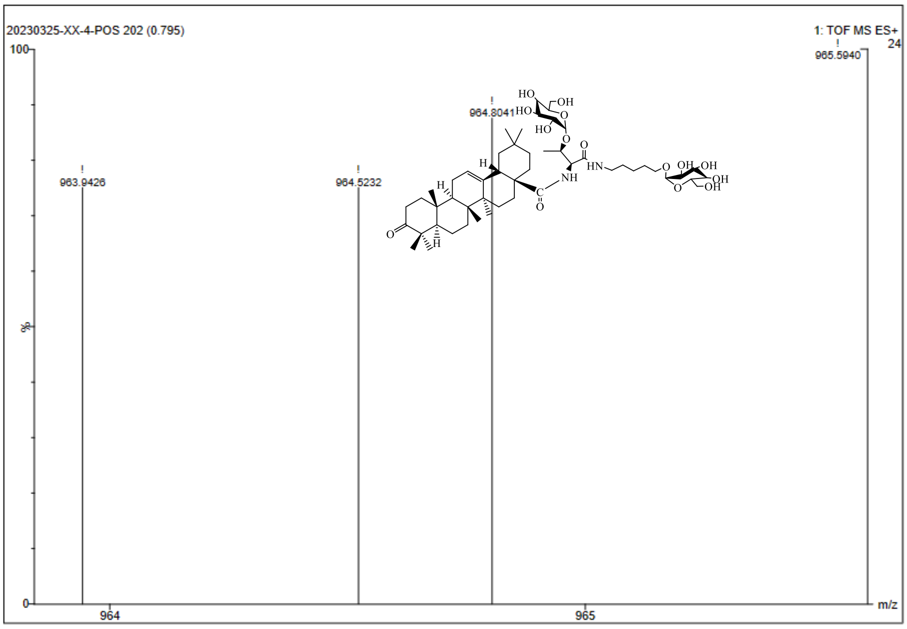

Figure S39. HRMS of L13 (ESI^+)

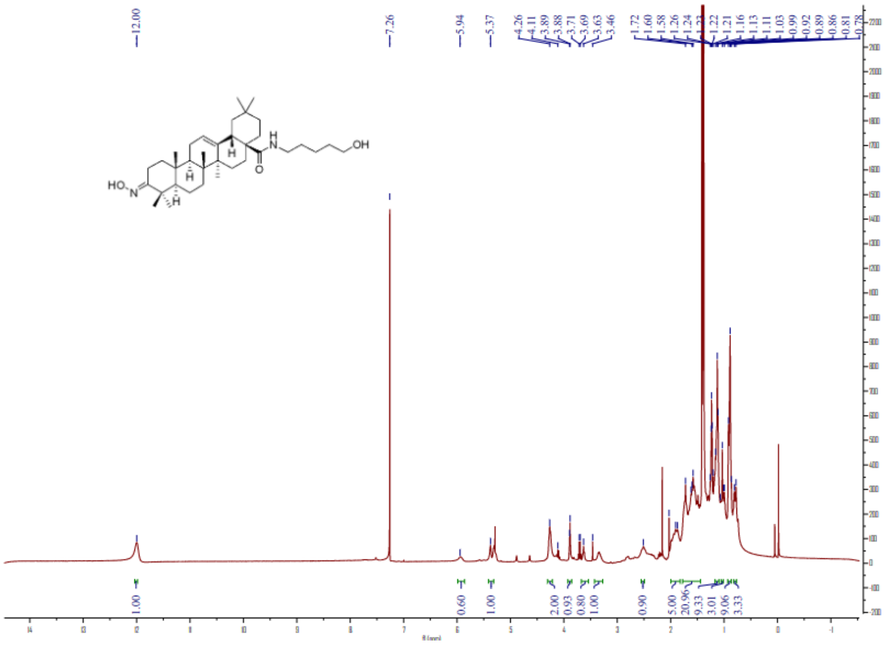

Figure S40. ^{1}H NMR of L14

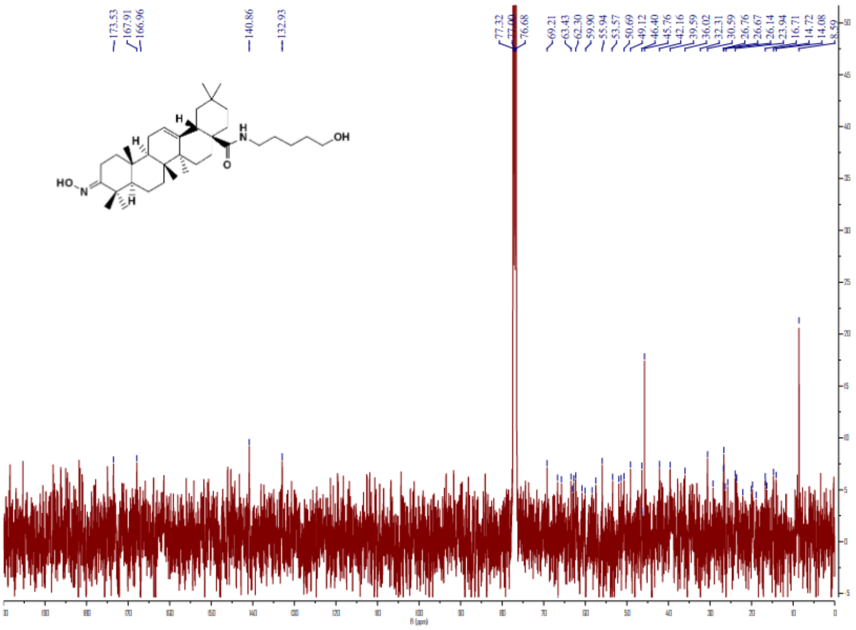

Figure S41. ^{13}C NMR of L14

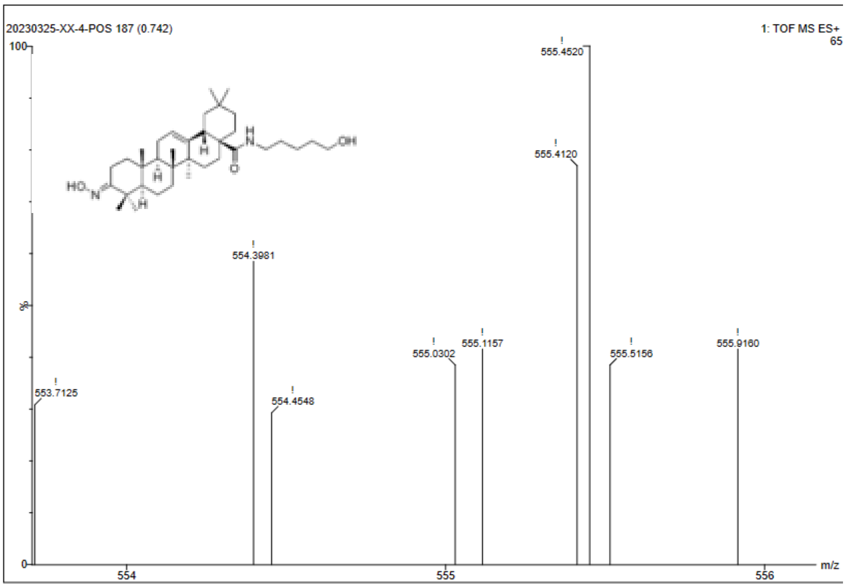

Figure S42. HRMS of L14 (ESI$^+$)

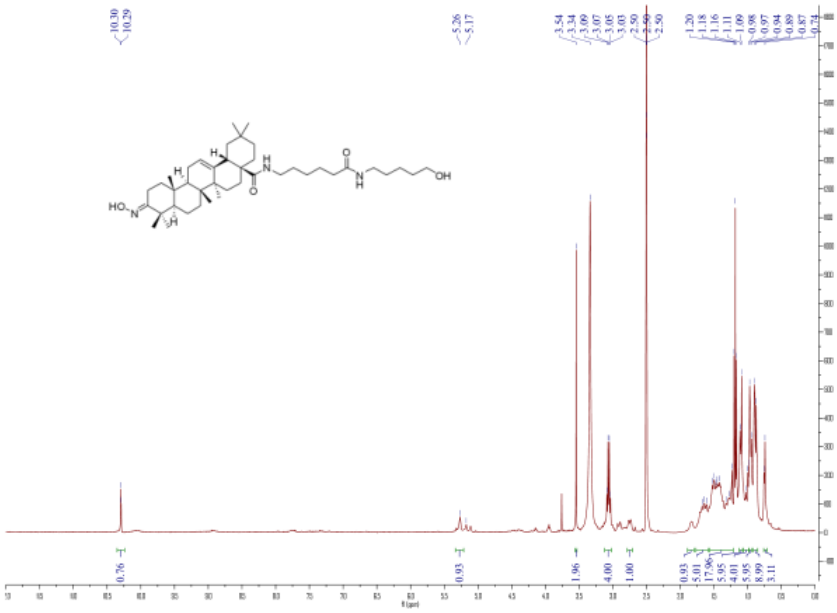

Figure S43. ¹H NMR of L15

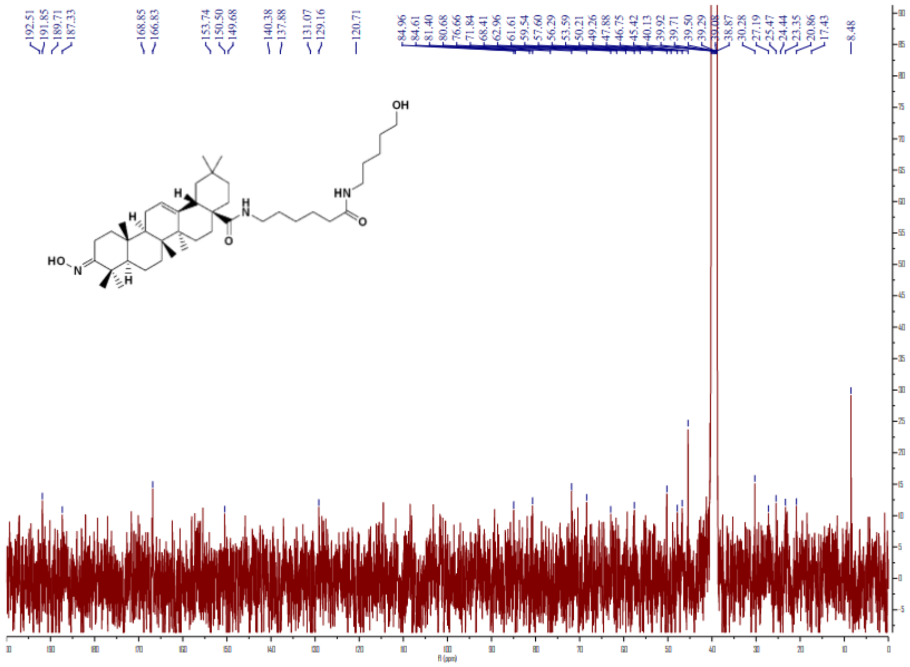

Figure S44. ¹³C NMR of L15

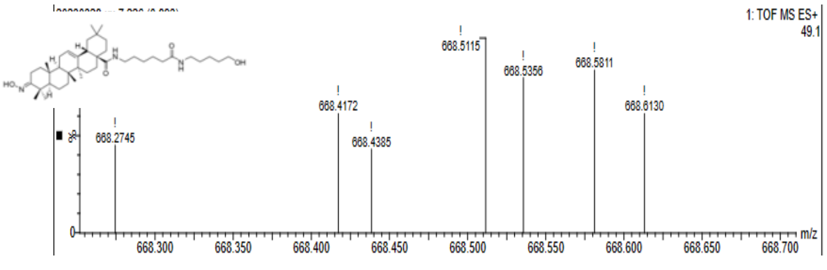

Figure S45. HRMS of L15 (ESI⁺)

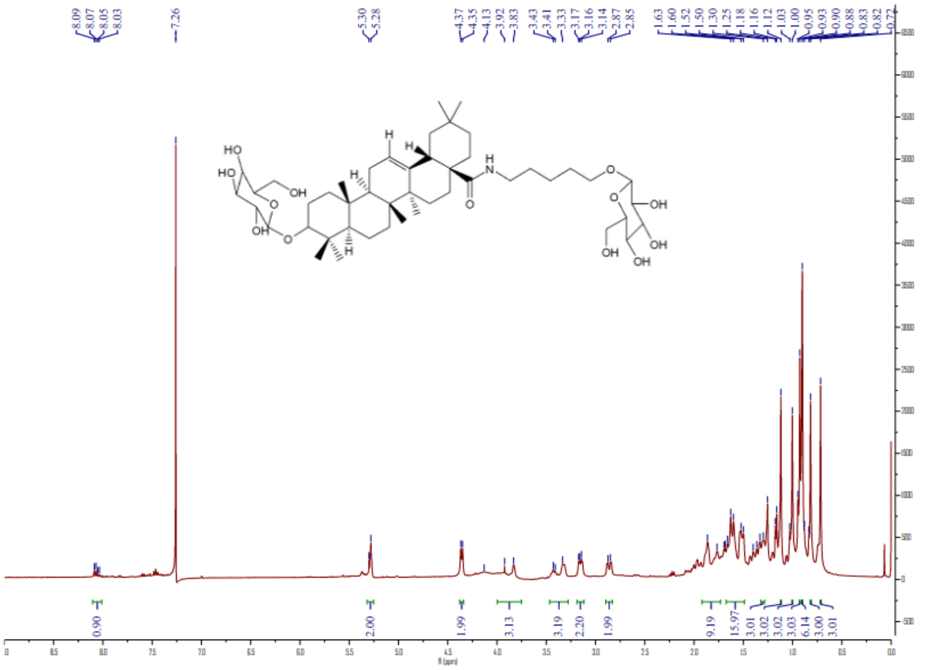

Figure S46. ^{1}H NMR of L16

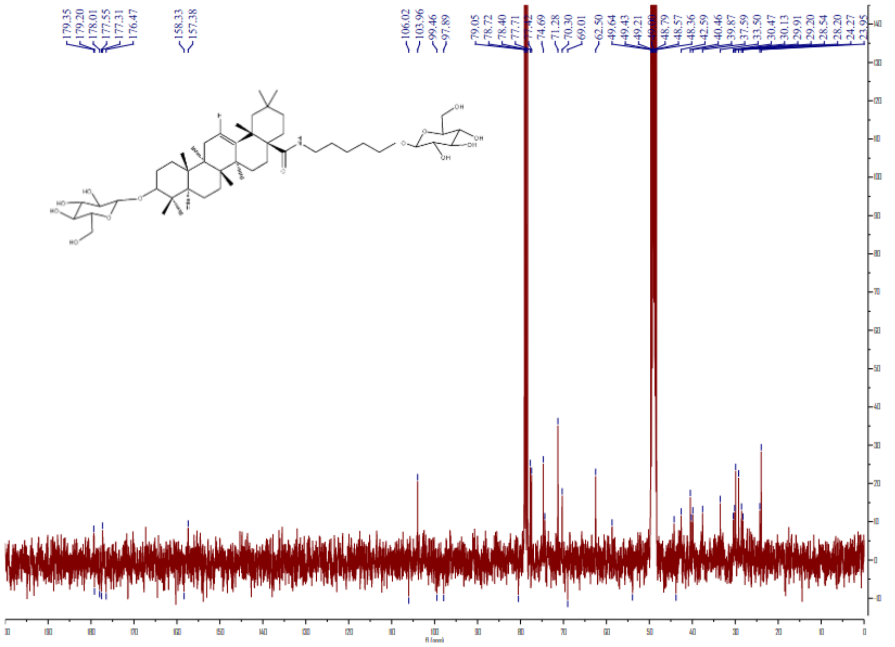

Figure S47. ^{13}C NMR of L16

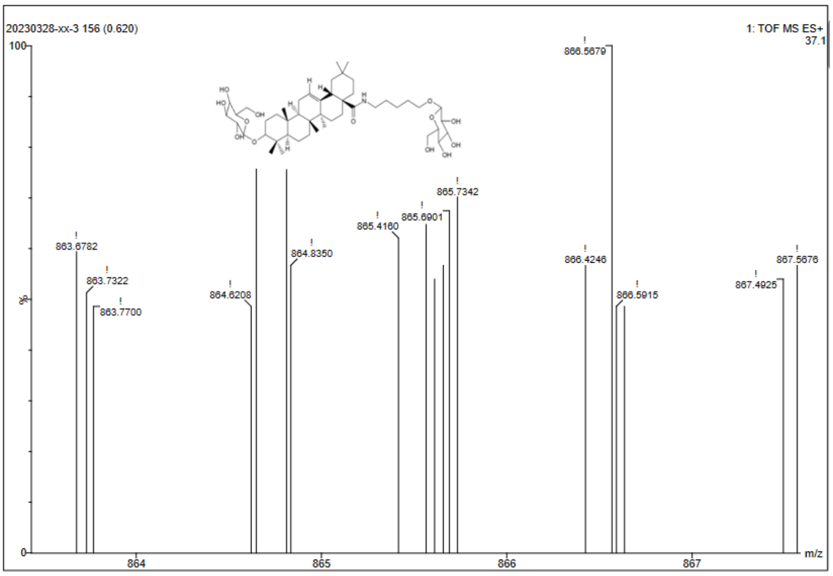

Figure S48. HRMS of L16 (ESI+)

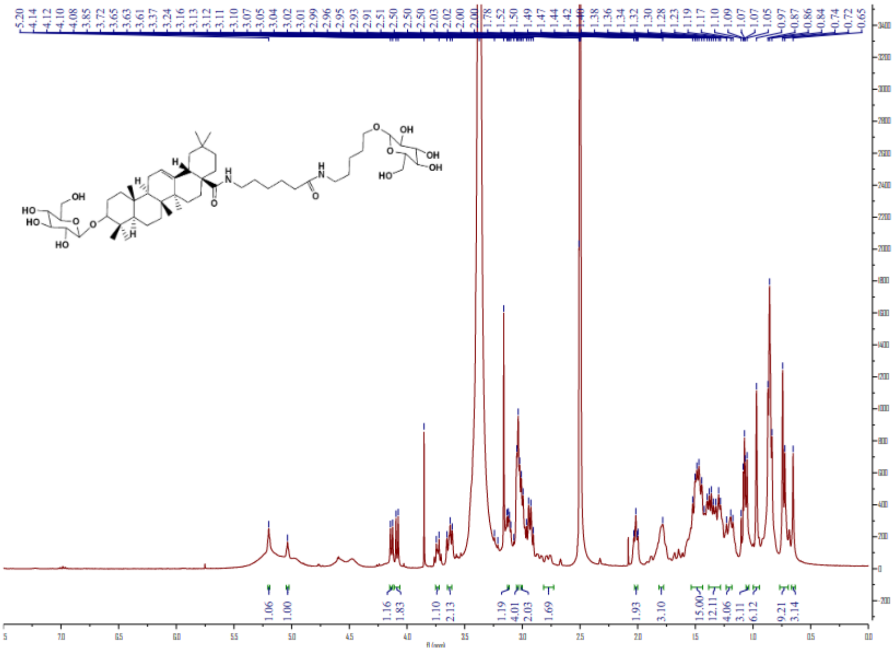

Figure S49. ^{1}H NMR of L17

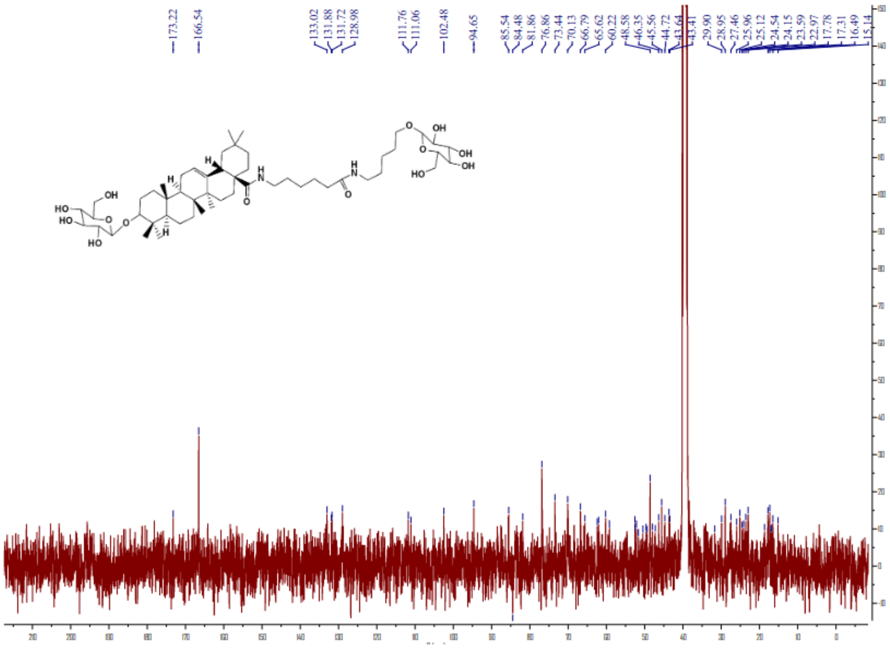

Figure S50. ^{13}C NMR of L17

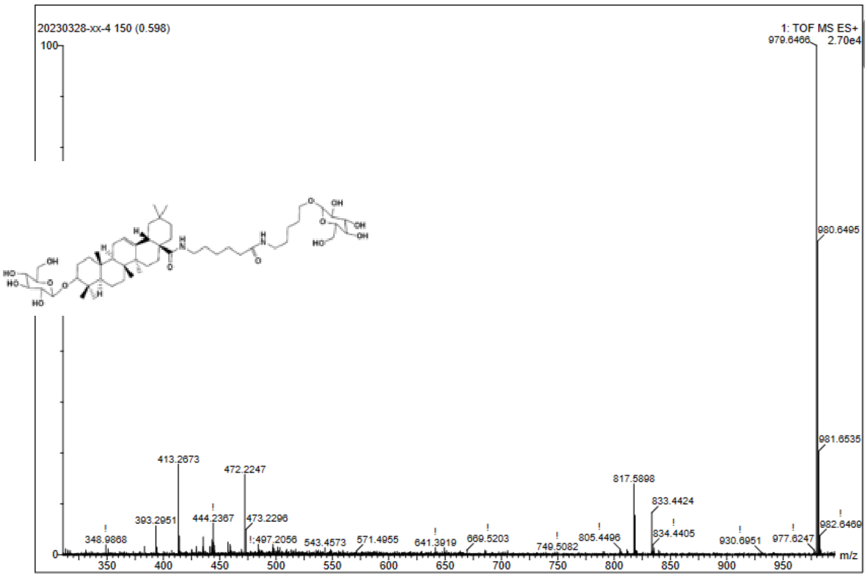

Figure S51. HRMS of L17 (ESI$^+$)

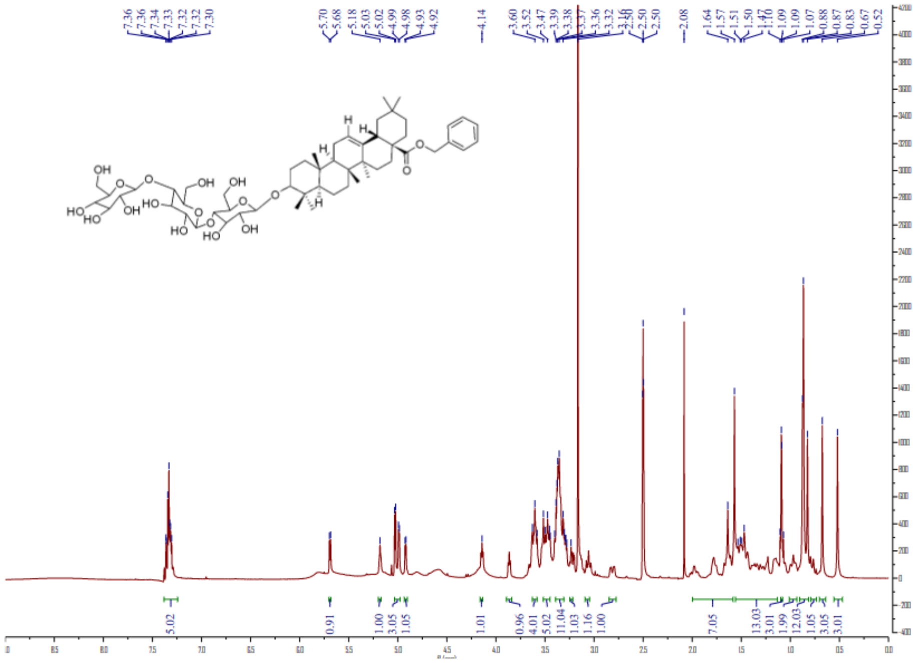

Figure S52. ^{1}H NMR of L18

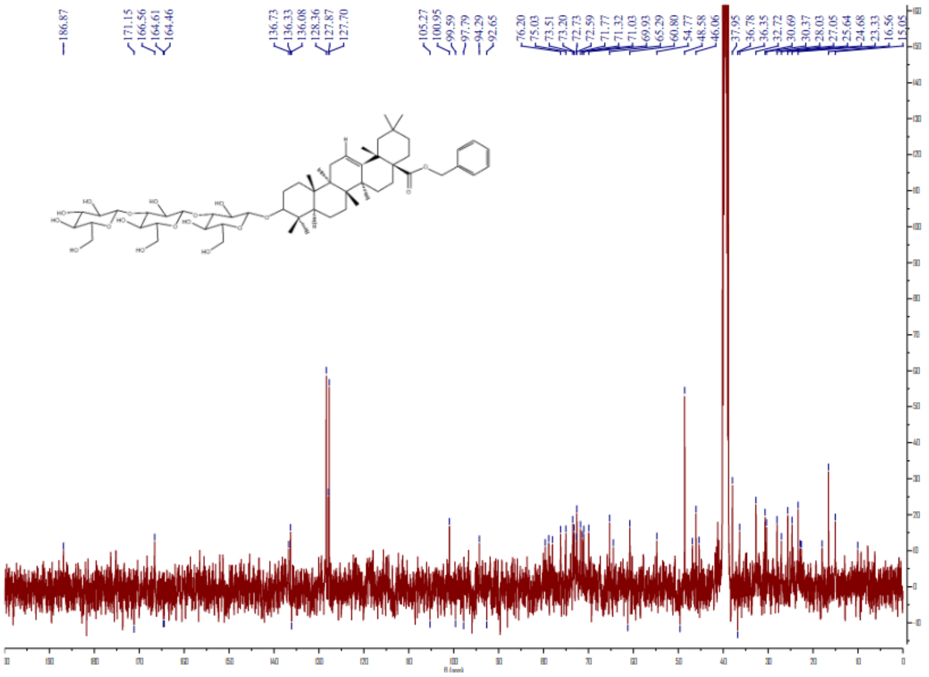

Figure S53. ^{13}C NMR of L18

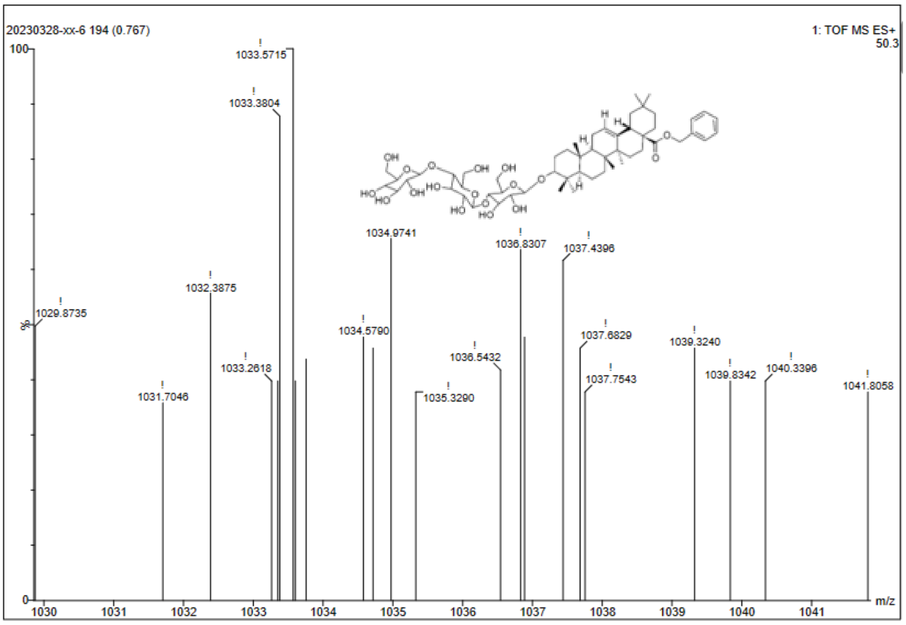

Figure S54. HRMS of L18 (ESI⁺)
